# RAF1 Expression is Correlated with HAF, a Parameter of Liver Computed Tomographic Perfusion, and may Predict the Early Therapeutic Response to Sorafenib in Advanced Hepatocellular Carcinoma Patients

**DOI:** 10.1515/med-2020-0024

**Published:** 2020-03-08

**Authors:** Ninzi Tian, Dong Wu, Ming Tang, Huichuan Sun, Yuan Ji, Cheng Huang, Lingli Chen, Gang Chen, Mengsu Zeng

**Affiliations:** 1 Department of Radiology, Zhongshan Hospital of Fudan University, 180 Fenglin Rd, Xuhui District, Shanghai 200032, China; 2 Shanghai Institute of Medical Imaging, Shanghai Medical College, Fudan University, Shanghai 200032, China; 3 Department of Liver Surgery, Zhongshan hospital of Fudan University, Shanghai 200032, China; 4 Department of Pathology, Zhongshan hospital of Fudan University, Shanghai 200032, China

**Keywords:** Hepatocellular carcinoma, Liver computed tomography perfusion, Sorafenib

## Abstract

**Objectives:**

Monitoring the early treatment effect of sorafenib in advanced hepatocellular carcinoma (HCC) patients is a diagnostic challenge. In a previous study, we reported the potential role of liver computed tomography perfusion (CTP) in the assessment of the response to sorafenib therapy in HCC. The present study aims to investigate whether sorafenib-targeted genes is correlated with CTP parameter, and investigate the potential of sorafenib-targeted genes in early prediction of therapeutic response to sorafenib in advanced HCC.

**Methods:**

A total of 21 HCC patients were enrolled. Sorafenib was administered orally at a dose of 400 mg twice daily continuously. Treatment response was assessed using modified response evaluation criteria in solid tumors (mRECIST) criteria. CTP scanning was performed before and after two weeks of sorafenib treatment using a 320-detector row CT scanner. The perfusion parameters of portal vein flow (PVF), hepatic artery flow (HAF), and perfusion index (PI) were acquired by CTP. The expression levels of several sorafenib-targeted genes were assayed using real-time quantitative PCR and western blot analysis. Logistic regression was performed to analyze the relationship between HAF values and RAF1 expression levels.

**Results:**

According to mRECIST, the disease control rate (CR+PR+SD) of treatment group was 70.5% after two months of treatment. Compared to background controls, tumor tissues exhibited higher HAF. A sorafenib-targeted gene, RAF1 expression, was increased in tumor tissues especially in the sorafenib-resistant group. The sorafenib-resistant group exhibited a significantly higher RAF1 expression and HAF than the sensitive group. Moreover, the RAF1 expression is positively correlated with the HAF value.

**Conclusion:**

RAF1 expression might predict therapeutic effects of sorafenib in advanced HCC, where RAF1 could potentially serve as a molecular marker for monitoring early therapeutic effects after sorafenib treatment.

## 1 Introduction

Sorafenib is the only standard treatment for advanced HCC to date, among the oral tyrosine kinase inhibitors, with a mean oral bioavailability 38-49% [1,2]. There exists an urgent need of precise evaluating approaches during the underway therapeutic period to report the early response to sorafenib, which may provide references to determine whether treatments should be continued and what alternative programs could be applied.

The present study investigates whether sorafenib-targeted genes is correlated with parameter of liver CT perfusion, and assessed the potential of sorafenib-targeted genes in early prediction of therapeutic response to sorafenib in advanced HCC.

## 2 Materials and methods

### 2.1 Patients

This was a retrospective study of a total of 21 patients with suspected liver tumors who underwent CT perfusion imaging between March 2014 to May 2016. This study was approved by the Ethics Committee of Zhongshan Hospital of Fudan University. Twenty-one patients were enrolled per the following inclusion criteria: (1) age of 18 years or older, (2) histologically confirmed primary HCC. The inclusion criteria were based on the Barcelona Clinic Liver Cancer (BCLC) standard. These patients were suggested to be treated with sorafenib. Sorafenib was administered orally at a dose of 400 mg twice daily continuously. When a clinician has to make a decision using pathological specimens, pathological samples were obtained by needle biopsy. Of the 21 patients, four patients ceased the sorafenib treatment for reasons of adverse reactions. We confirmed that sorafenib-responders and non-responders have no differences in all regular clinical features (sex, age, origin of liver diseases, etc.) (Table.1).

**Table 1:** Patient’s main characteristics.

Clinical Features	Advanced HCC with drug resistance(n=12)	Advanced HCC with drug effciency(n=5)	P value
Age	Mean: 56.3 years (range 50.2–62.7)	Mean: 57.2 years (range 48.6–68.5)	0.632
Sex (%)			0.441
Male	7	3	
Female	5	2	
Origin of liver disease (%)			1.000
Hepatitis B	12	4	
Other	0	1	
History of hepatitis B (%)			0.716
≤3 y	2	1	
10–30 y	10	4	
>30 y	0	2	
RAF level			0.038
Higher expression	3	4	
Lower expression	7	1	

Informed consent has been obtained from all individuals included in this study

### 2.2 Computed tomography perfusion (CTP) scanning

CTP was performed on an average two week before treatment and on two weeks after treatment using a 320-slice multi-detector CT scanner (Aquilion ONEViSION; Toshiba Medical Systems Corporation, Otawara, Japan). CTP was performed using the dynamic volume scan mode with 16 cm z-axis coverage and no table movement. Before scanning, 0.5ml/kg of nonionic iodinated contrast medium was intravenously injected (Iopamiron 370; Bayer Health Care, Guangdong, China), followed by 30 mL of saline solution using a power injector at a rate of 8 mL/sec through a 18-gauge cannula placed in the antecubital vein. The CTP acquisition protocol was initiated simultaneously with the start of the contrast injection. The first volume acquisition took place 8 seconds after the contrast administration. All patients were asked to breathe gently during the entire acquisition, and restraining bands were placed around the abdomen to limit respiration movement of the abdomen. A total scan duration of 74 seconds was separated into three phases: the first 11 volumes were acquired every 2 second, followed by 7 volumes during every 3 seconds, and 5 volumes during every 5 seconds. Each patient was exposed to the x-ray for 6.9 seconds. All volumes were acquired with the following parameters: 100 kVp, 100 mA, and 0.3 second rotation speed. Each volume was reconstructed at a 0.5 mm thickness with 0.5 mm spacing providing a total of 7360 (23 volumes × 320 images) images.

### 2.3 CTP analysis

Body Perfusion software (Vitrea v6.5.3, Toshiba Medical Systems Corporation, Otawara, Japan) was used for CTP analysis. Prior to perfusion analysis, deformation registration was performed on the workstation to take into account the volume mismatch between the volumes. The entire registration process takes about 3 minutes for a single check. The analysis applied the dual-input maximum slope method. To generate time-density curves, the region of interests (ROIs) was placed on the abdominal aorta at the level of celiac axis, main portal vein, liver, and spleen. Then, functional maps were automatically generated, representing each pixel value of hepatic artery flow (HAF, mL/100ml/minute), portal vein flow (PVF, mL/100 ml/minute), and perfusion index (PI expressed as a percentage) using a color scale.

The perfusion maps were then viewed in 5mm slice thickness. HAF, PVF, and PI were measured in the tumor and in an area with background liver tissue (no tumor area) in each patient by two radiologists who had more than 16 years of experience in abdominal imaging (W.D. and T.M.). A tissue time-enhancement curve as well as colored functional maps of HAF, PVF, and PI were automatically derived for selected ROIs. HAF, PVF, and PI of tumor and normal liver tissue were automatically calculated respectively.

### 2.4 Quantitative real-time PCR (qPCR)

QPCR was performed to determine the expression of RAF1, VEGFR, PDGFR-B, and FLT3 mRNA. Briefly, total RNA was extracted from tumor tissue and adjacent normal control using Trizol Reagent (Invitrogen, Carlsbad, CA, USA). The reverse transcription (RT) for mRNA was carried out using the Oligo dT primer. qPCR was carried out on Applied Biosystems 7300 real-time PCR system (Applied Biosystems, Foster City, CA) using a standard protocol from the SYBR Green PCR kit (Toyobo, Osaka, Japan). The sequences of primers were as follows: Raf-1 Forward: 5′-CGCTTAGATTGGAATACTGA-3′, Reverse: 5′-AAAGGTGAAGGCGTGAG-3′; VEGF-R Forward: 5-AACGTGTCACTTTGTGCAAGA-3′Reverse: 5′-TTCCATGAGACGGACTCAGAA-3′; PDGFR-b Forward: 5′-TGATGCCGAGGAACTATTCATCT-3′, Reverse: 5′-TTTCTTCTCGTGCAGTGTCAC-3′;

Flt3 Forward: 5′-CGGGCTCACCTGGGAATTAG-3′, Reverse: 5′-GTCGTTTCTTGCCACTGATGA-3′; GAPDH Forward: 5′-GCCACATCGCTCAGACAC-3′

Reverse: 5′-CATCACGCCACAGTTTCC-3′. All PCRs were performed in triplicates. Relative quantification mRNA expression was calculated using the 2^−ΔΔCT^ method.

### 2.5 Western blot

Equivalent amounts of protein (80 μg) were electrophoresed through a 15% SDS-polyacrylamide gel, and then wet electro-transferred to 0.2 μm PVDF membranes (Bio-Rad, CA, USA). The blots were incubated overnight at 4°C with antibodies (Cat # ab137435, USA), followed by incubation with a goat anti-rabbit HRP-conjugated secondary antibody (1:5000, Jackson, USA). Protein signals were visualized by enhanced chemiluminescence detection (Pierce Biotechonology, Rockford, IL). Actin was used as loading control.

### 2.6 Statistical analysis

As the quantitative data could fit the normal distribution, t-test and one-way ANOVA was applied for comparison. The final data was expressed as mean ± SE; and a value of P <0.05 was considered statistically significant. Multivariate logistic regression was performed to analyze the relationship between all the CT perfusion features and RAF1 expression levels.

## 3 Results

### 3.1 Tumor tissues exhibited higher HAF in the group that responded to sorafenib

**Figure 1:**
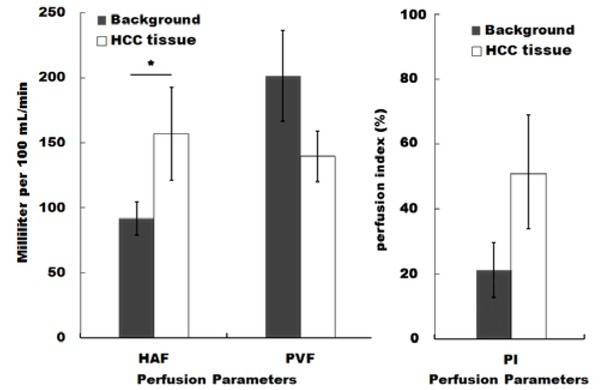
Hepatocellular carcinoma (HCC) patients exhibited higher intrahepatic blood flow and perfusion index. Parameters including hepatic artery flow (HAF), portal vein flow (PVF), and perfusion index (PI) are presented as mean±SE. * p<0.05 HCC vs control.

**Figure 2:**
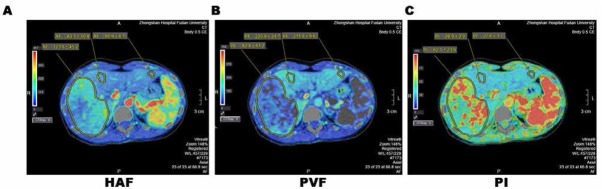
A 37-year-women was diagnosed as advanced HCC. A to C, Liver perfusion CT maps of HAF, PVF, and PI, respectively. The backrground liver parenchyma HAF was 83.3±10.8 and 80.9±6.1mL per 100 mL/min, HCC PVF was 123.0±45.2 mL per 100 mL/min.

Parameters including HAF, PVF, and PI were obtained using CTP scanning. As shown in Table 2 and Figure 1 and 2, tumor tissues showed significantly higher levels of HAF compared to the background controls, and the increase in HAF parameters in tumor is mainly contributed by the sorafenib-resistant groups (Table 3). Whereas the change of PVF or PI in tumor tissues was not significant compared to control. These data suggest that tumor tissues have differential HAF levels compared to background control, and tumor tissues in sorafenib responder group exhibited lower HAF compared to tumor tissues in non-responder group.

**Table 2:** HAF,PVF and PI obtained by pertusion CTP scanning

	Parameters	MEAN	SD	Minimum	Maximum	P value
Background Liver	HAF	78.3	10.8	68.4	89.3	
	PVF	171.3	29.6	142.6	230.6	
	PI	20.3	7.7	15.3	29.6	
	HAF	135.5	35.6	96.9	188.4	0.039
HCC	PVF	118.9	16.7	81.9	133.4	0.087
	PI	48.9	17.6	28.5	67.2	0.108

HAF indicates hepatic artery flow (milliliter per 100 mL/min); PI, perfusion index (%); PVF, portalvein flow (milliliter per 100 mL/min)

**Table 3:** Parameters for patients before treatment at CR+PR+SD and PD group according to RECIST progression

Parameters		CR+PR+SD(n=12)	PD(n=5)	P value
HAF		90.1±17.8	128.8±13.6	0.037*
PVF		161.9±32.3	154.6±21.1	0.129
PI		48.9±10.7	49.3±9.8	0.161

HAF indicates hepatic artery flow (milliliter per 100 mL/min); PI, perfusion index (%); PVF, portal vein flow (milliliter per 100 mL/min)

### 3.2 RAF1 expression was increased in tumor tissues especially in the sorafenib-resistant group

**Figure 3:**
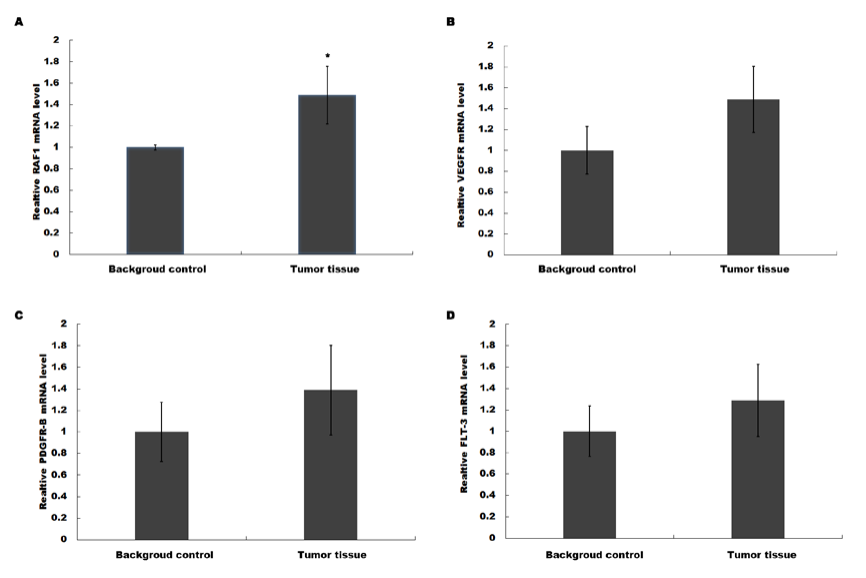
RAF1 expression was increased in HCC patients. Several widely recognized oncogenes were determined by Realtime qPCR, including RAF1 (A), FLT-3 (B), VEGFR (C) and PDGFR-B (D), which were commonly regarded as useful targets of sorafenib. RAF1 mRNA level showed a highly significant enhancement. ** p<0.01 HCC vs control.

**Figure 4:**
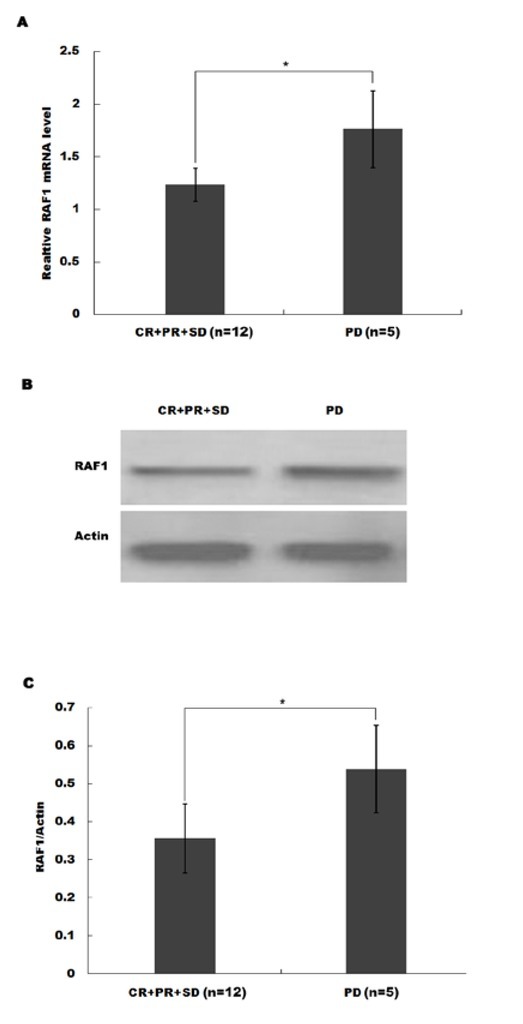
The resistant sub-group (SD) had even higher expression of RAF1 mRNA (A) and protein (B-C) compared to the sensitive subgroup (CR+PR+SD). The HCC patients were divided into four populations according to different responses to sorafenib, which were the complete response (CR) group, the partial response (PR) group, the stable disease (SD) group, and the progressive disease (PD) group. ** p<0.01 HCC vs control.

Next, we attempted to probe potential relationships between CTP parameters and sorafenib-targeted oncogenes. Several widely recognized oncogenes were determined by qPCR, including RAF1, FLT-3, VEGFR, and PDGFR-B, which were commonly regarded as useful targets of sorafenib. As shown in Figure 3, only RAF1 mRNA level showed a significant increase in tumor tissues compared background control. We further confirmed the protein expression of RAF1 through Western blot, after the HCC group was divided into two sub-groups according to different responses to sorafenib, which were the complete response (CR), the partial response (PR), the stable disease (SD), and the progressive disease (PD). The sorafenib resistant sub-group (PD) had even higher expression of RAF1 mRNA (Figure.4A) and protein (Figure.4B-C) compared to the sensitive sub-group (SD+CR+PR). Consequently, RAF1 might be a molecular marker for HCC progressiveness or drug resistance.

### 3.3 HAF positively correlates with the RAF1 expression

**Figure 5:**
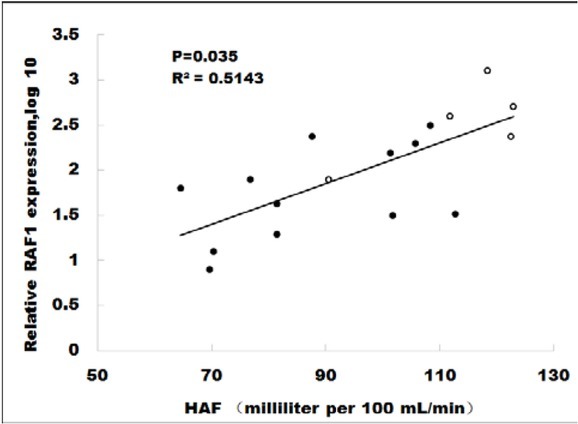
HAF value correlates with the RAF1 expression. Logistic regression showed a significantly positive linear correlation between HAF values and RAF1 expression levels. Moreover, the sorafenib-resistant samples (blue dots) demonstrated higher levels of HAF and RAF1 expression.

Finally, we performed logistic regression to analyze the relationship between HAF values and RAF1 expression levels, and found a significantly positive linear correlation (Figure.5, p=0.035, R^2^=0.5143). Moreover, the sorafenib-resistant samples (blue dots) demonstrated higher levels of HAF and RAF1 expression. These findings highlight that HAF could be an early predictor for the response to sorafenib. Those patients with a high HAF value are likely to carry an overexpressed RAF1 level, and thus end up with a poor prognosis.

## 4 Discussion

In the present study, we demonstrated that sorafenib resistant patients had higher RAF1 and HAF levels than sensitive. Moreover, the HAF level had a positive correlation with the RAF1 expression, which implied that the RAF1 level may serve as an early diagnostic marker for the final outcomes of sorafenib administration.

Sorafenib is so far regarded the foremost treatment available for advanced HCC [3]. For patients, there are few lack effective parameters to observe when assessing the treatment effects of sorafenib, given significant shrinkage may not be easily detected especially in the early therapy stage. It is vital of a precise evaluation of the early therapeutic response to determine whether the treatment has been effective and should be recommended to continue.

To date, the guideline of sorafenib selection was mainly in accordance with the RECIST 1.1. However, this criterion is limited in reflecting the cell activity, and hemodynamic changes. Therefore, RECIST V1.1 could not fully meet the needs of predicting the progression of HCC. Perfusion CT has been widely used in the clinical examination of human diseases, including cardiovascular disease, stroke, and cancer [4,5]. Perfusion CT scanning carries out quantitative assessment of the changes in the blood flow of the microenvironment. Given that perfusion imaging provides the ability to detect regional alterations in organ blood flow, it is sufficiently reasonable to discover novel parameters in CT data [6,7]. The HAF level is a widely agreed index for assessment of hepatic pathological changes and malignancy [8], even a potential ideal guideline to distinguish between pre-carcinoma and early HCC nodules [9].

There exist urgent needs to improve standards of receiving sorafenib treatment for advanced HCC. Accumulated researches have suggested that there exist all kinds of molecular markers (including signaling activation levels and expression of genes or microRNAs) [10, 11, 12] potentially valuable for prediction of sorafenib response, but the corresponding clinical applications at present have still been far from satisfactory [13,14]. Accordingly, there is a growing need using novel non-invasive imaging methods to accurately evaluate the later therapeutic effects. Some scholars have started to take advantages of similar approaches to the present study. For example, it has recently been reported about the diagnostic values of intra-voxel incoherent motion imaging for monitoring the therapeutic response of sorafenib on HCC in mouse xenograft models [15]. More intriguingly, a similar study also applied perfusion CT in the quantitative assessment of response to sorafenib in advanced HCC patients. The perfusion values HAF and PI were significantly higher in HCC than cirrhotic parenchyma, and the sensitive group showed a reduction of CT values after therapy while the resistant group demonstrated no variation before and after treatment [16]. However, their study had not observed a predictive value of the CT parameters (HAF in particular) for the later response to sorafenib, although their conclusion and ours could support each other mutually. Another study subjected the HCC mouse model to elastosonography and claimed that elastosonography might be a promising noninvasive technique for the early prediction of sorafenib response [17]. Those with good treatment response showed an increase in elasticity on day + 2 while the others showed a decrease in elasticity. In the future, combination of all validated noninvasive predictors may become a routine diagnostic procedure.

The HAF level is a widely agreed index for microcirculation hemodynamics changes. One research noted the perfusion parameters BF, HAF, and IRFTO could be used to determine the hepatic malignancy [8]. Another study pointed out that CT perfusion combined with ROC curve analysis is a new diagnosis model for distinguishing between pre-carcinoma and early HCC nodules, in which the HAF level is an ideal guideline [9]. Statistically, most data agree that HCC patients have an increased HAF. Enhanced HAF indicates reduced portal vein blood supply while increased arterial blood supply due to intrahepatic lesions, which strongly support the diagnosis of malignant tumors. In our correlation analysis, a higher level of HAF not only meant a greater likelihood of malignant case, but also a higher possibility of sorafenib resistance. The patients with highest levels of HAF were almost sorafenib resistant ones in late treatment. A possible mechanism may be due to a higher HAF indicates more difficulties for drug arrival in the lesion, and thus weaker response. To date, this study is the first one discovering a predictive value of HAF to forecast sorafenib response in HCC patients. Still, more evidences are merited to confirm the effectiveness.

RAF1 is a widely recognized oncogene. As a MAP kinase (MAP3K), it activates the downstream ERK signaling pathway and consequently promotes cell proliferation. Independent studies have proved the expression of RAF1 was increased in HCC [18,19]. In addition, RAF1 is also one of the targets or mechanisms of sorafenib, which inhibits the Raf1/c-Raf serine/threonine kinase phosphorylation in the MAPK pathways [20]. However, there exists only one striking study which unexpectedly discovered a RAF1 expression reduction in human HCC samples. In this study, RAF1 downregulation increased the proliferation of HCC in xenografts and in culture [21]. Our conclusion seems to point that highly expressed RAF1 is associated with sorafenib resistance. This makes sense theoretically, but the realities are much more complicated. Some observed influence of sorafenib on proliferation was not simply through the RAF cascade, for the responses of RAF1 to sorafenib among individuals were diverse, sometimes even contrary [22]. Overall, the sorafenib resistant tumors may develop other strategies to activate RAF1 and consequently its downstream effectors, for example MRP3 [23,24]. Using CT scan could alert to target other RAF1 relating pathways in advance before a regular sorafenib administration. In conclusion, we here propose a new diagnostic marker, the expression level of RAF1, to predict the early treatment effects of sorafenib on advanced liver cancer.

The limitations of the current study lie in the following aspects: i) The sample size is small. It is necessary to verify the accuracy of the correlation of HAF with RAF1 in larger sample sizes. ii) Our software only permits the use of ROIs drawn in a single image plane (in which the tumor diameter was maximal); it was not possible to use volumes of interest for the analysis of perfusion parameters, which would have represented a more robust approach. iii) Variations in the degree of the disease condition between the study participants may also have had some effect on the results. Hence, further research with larger sample sizes are required to validate the study results.

## References

[1] Liu C, Chen Z, Chen Y, Lu J, Li Y, Wang S (2016). Improving Oral Bioavailability of Sorafenib by Optimizing the “Spring” and “Parachute” Based on Molecular Interaction Mechanisms. Mol Pharm.

[2] Sugimoto K, Moriyasu F, Saito K, Rognin N, Kamiyama N, Furuichi Y (2013). Hepatocellular carcinoma treated with sorafenib: early detection of treatment response and major adverse events by contrast-enhanced US. Liver international: official journal of the International Association for the Study of the Liver.

[3] Gang G, Hongkai Y, Xu Z (2015). Sorafenib combined with radiofrequency ablation in the treatment of a patient with renal cell carcinoma plus primary hepatocellular carcinoma. Journal of cancer research and therapeutics.

[4] Wintermark M (2005). Brain perfusion-CT in acute stroke patients. European radiology.

[5] Tatli S, Lipton MJ (2005). CT for intracardiac thrombi and tumors. The international journal of cardiovascular imaging.

[6] Joshi PV, Lele VR, Bhat GM, Garg S, Chitale A (2012). F-18 flourodeoxyglucose positron emission tomography/computed tomography findings in a case of hepatosplenic T-cell lymphoma. Journal of cancer research and therapeutics.

[7] Yoganathan SA, Maria Das KJ, Subramanian VS, Raj DG, Agarwal A, Kumar S (2017). Investigating different computed tomography techniques for internal target volume definition. Journal of cancer research and therapeutics.

[8] Singh J, Sharma S, Aggarwal N, Sood RG, Sood S, Sidhu R (2014). Role of Perfusion CT Differentiating Hemangiomas from Malignant Hepatic Lesions. J Clin Imaging Sci.

[9] Li JP, Feng GL, Li DQ, Wang HB, Zhao DL, Wan Y (2016). Detection and differentiation of early hepatocellular carcinoma from cirrhosis using CT perfusion in a rat liver model. Hepatobiliary Pancreat Dis Int.

[10] Nishida N, Arizumi T, Hagiwara S, Ida H, Sakurai T, Kudo M (2017). MicroRNAs for the Prediction of Early Response to Sorafenib Treatment in Human Hepatocellular Carcinoma. Liver Cancer.

[11] Vaira V, Roncalli M, Carnaghi C, Faversani A, Maggioni M, Augello C (2015). MicroRNA-425-3p predicts response to sorafenib therapy in patients with hepatocellular carcinoma. Liver Int.

[12] Zhu AX (2009). Predicting the response to sorafenib in hepatocellular carcinoma: where is the evidence for phosphorylated extracellular signaling-regulated kinase (pERK)?. BMC Med.

[13] Shomura M, Kagawa T, Shiraishi K, Hirose S, Arase Y, Koizumi J (2014). Skin toxicity predicts efficacy to sorafenib in patients with advanced hepatocellular carcinoma. World J Hepatol.

[14] Howell J, Pinato DJ, Ramaswami R, Bettinger D, Arizumi T, Ferrari C (2017). On-target sorafenib toxicity predicts improved survival in hepatocellular carcinoma: a multi-centre, prospective study. Aliment Pharmacol Ther.

[15] Lee Y, Lee SS, Cheong H, Lee CK, Kim N, Son WC (2017). Intravoxel incoherent motion MRI for monitoring the therapeutic response of hepatocellular carcinoma to sorafenib treatment in mouse xenograft tumor models. Acta Radiol.

[16] Ippolito D, Querques G, Okolicsanyi S, Franzesi CT, Strazzabosco M, Sironi S (2017). Diagnostic value of dynamic contrast-enhanced CT with perfusion imaging in the quantitative assessment of tumor response to sorafenib in patients with advanced hepatocellular carcinoma: A feasibility study. Eur J Radiol.

[17] Salvatore V, Baron Toaldo M, Marinelli S, Milazzo M, Palama C, Venerandi L (2013). Early prediction of treatment response to sorafenib with elastosonography in a mice xenograft model of hepatocellular carcinoma: a proof-of-concept study. Ultraschall Med.

[18] Qu J, Li J, Chen K, Qin D, Li K, Sheng Y (2013). Hepatitis B virus regulation of Raf1 promoter activity through activation of transcription factor AP-2alpha. Arch Virol.

[19] Tian Y, Hu Y, Wang Z, Chen K, Zhang L, Wang L (2011). Hepatitis B virus regulates Raf1 expression in HepG2.2.15 cells by enhancing its promoter activity. Arch Virol.

[20] Gauthier A, Ho M (2013). Role of sorafenib in the treatment of advanced hepatocellular carcinoma: An update. Hepatol Res.

[21] Jeric I, Maurer G, Cavallo AL, Raguz J, Desideri E, Tarkowski B (2016). A cell-autonomous tumour suppressor role of RAF1 in hepatocarcinogenesis. Nat Commun.

[22] Lin ZY, Chuang WL (2017). Contrary influence of clinically applied sorafenib concentrations among hepatocellular carcinoma patients. Biomed Pharmacother.

[23] Lin S, Hoffmann K, Xiao Z, Jin N, Galli U, Mohr E (2013). MEK inhibition induced downregulation of MRP1 and MRP3 expression in experimental hepatocellular carcinoma. Cancer Cell Int.

[24] Tomonari T, Takeishi S, Taniguchi T, Tanaka T, Tanaka H, Fujimoto S (2016). MRP3 as a novel resistance factor for sorafenib in hepatocellular carcinoma. Oncotarget.

